# Walkie talkies to aid health care workers’ compliance with personal protective equipment in the fight against COVID-19

**DOI:** 10.1186/s13054-020-03150-8

**Published:** 2020-07-13

**Authors:** Dominic Fenn, Jonny Coppel, Jessica Kearney, Lucy Everson, Simon Braithwaite, Rahul Chodhari

**Affiliations:** 1 Asthma Innovation Research, London, UK; 2 Department of Respiratory Medicine, Amsterdam UMC, Amsterdam, The Netherlands; 3 Department of Intensive Care, Royal Free Hospital, Barnet site, London, UK; 4 Department of Medicine, Kings College Hospital, London, UK; 5 Department of Obstetrics and Gynecology, Southmead Hospital, Bristol, UK; 6 Department of Radiology, John Radcliff Hospital, Oxford, UK; 7 Department of Paediatrics, Royal Free Hospital, London, UK

Dear Editor

The recent article by Houghton et al. in the Cochrane Database of Systematic Reviews highlighted some key limitations associated with health care workers’ compliance with infection prevention and control (IPC) guidelines [1]. Addressing barriers highlighted by this review is crucial to keep healthcare workers and patients safe during the SARS-CoV-2 pandemic.

They identify that the practicalities of donning and doffing personal protective equipment (PPE) are a barrier to adherence. They recognise this process as time-consuming and detrimental to healthcare workers’ health [1]. However, we wanted to draw the readers’ attention to another barrier created by PPE. Whilst wearing full PPE, communication between COVID and non-COVID designated areas becomes a challenge [2]. Firstly, phones and pagers are difficult to access or cannot be used due to contamination risk. Secondly, staff must remove PPE when moving from one clinical area to another. This both delays communication and serves as an additional drain on precious PPE resources [1].

What can be done? One approach is to use walkie talkies. Liew et al. draw attention to their use in Singapore [3]. They provide a quick, resource-efficient and effective means of communicating and may provide a solution. At our charity, Asthma Innovation Research (AIR), we recognised the potential of walkie talkies through the team’s personal experience and have subsequently supplied over 75 hospitals across the UK. Nevertheless, while they have several attributes that make them suitable for use during the current pandemic, there are limitations that must be acknowledged and addressed (Tables 1 and 2). For example, one of the most attractive advantages is also the biggest shortcoming: Commercially available walkie talkies are licence-free and are not dependent on pre-existing telecommunication infrastructure. Therefore, they can be rapidly deployed with no additional costs and allow for timely integration into practice. However, this makes them vulnerable to data breach as the channel may be disrupted by non-health care professionals. Thankfully, awareness of this issue and proactive mitigation can significantly reduce this risk (Table 2). Although walkie talkies may not be a perfect system, they provide a much needed immediate solution. However, preparations for further pandemics should be made, addressing these limitations (Table 2).

**Table 1:** Advantages and disadvantages of walkie talkie use in health care settings

Advantages	Disadvantages
Low cost	Non-secure channel
Easy to decontaminate per IPC guidance	Miscommunication between multiple teams sharing the same channel
Simple to operate	Radio frequencies may be obstructed by hospital structures (e.g. lead lining within radiotherapy departments)
Can be used while wearing PPE	Specific communication approach required
Does not rely on telecommunication infrastructure	
“One to many” communication provides instant access to the whole team

**Table 2:** Current and future solutions to key disadvantages of walkie talkie use in health care settings

Issue	Current solution	Future solutions
Non-secure channel	- Avoid patient identifiable information - Frequency hopping: a coordinated channel switch by the team arranged off an open channel	- Investment into secure radio systems that use: Encrypted frequencies Automatic frequency-hopping
Miscommunication between multiple teams sharing the same channel	- If more than one different department using walkie talkies, department heads should establish which channel to use for each team to keep communications separate.	
Radio frequencies may be obstructed by hospital structures (e.g. lead lining within radiotherapy departments)	- To be aware of signal loss zones and make sure alternative means of communication are available	- Increased transmission power of radio (requires listened frequencies) - Use of radio repeater
Specific communication approach required	- Communication should be clear, structured and succinct. It must be obvious who you are and who you are trying to contact. For example: *“Person A: This is Person A calling Person B, come in Person B, over …*.” *“Person B: This is Person B receiving over … ..”*	- Teaching on use of walkie talkie communication and etiquette as part of departmental staff induction - Simulation training to incorporate walkie talkies

Adherence to correct IPC guidance is crucially important during the current pandemic, in addition to effective communication. Walkie talkies are a potential method of enabling timely, coordinated and safe care for patients, while also protecting patients and staff.

## Data Availability

Not applicable
